# The clinical and biochemical effectiveness and safety of cholic acid treatment for bile acid synthesis defects: a systematic review

**DOI:** 10.1186/s13023-024-03449-7

**Published:** 2024-12-19

**Authors:** Yasmin Polak, Laura van Dussen, E. Marleen Kemper, Frédéric M. Vaz, Femke C. C. Klouwer, Marc Engelen, Carla E. M. Hollak

**Affiliations:** 1 Department of Pharmacy and Clinical Pharmacology, Amsterdam UMC, University of Amsterdam, Amsterdam, The Netherlands; 2 Department of Endocrinology and Metabolism, Amsterdam UMC, University of Amsterdam, Meibergdreef 9, Amsterdam, 1105 AZ The Netherlands; 3 Department of Pediatric Neurology, Amsterdam UMC, University of Amsterdam, Amsterdam, The Netherlands; 4 Department of Laboratory Medicine and Pediatrics, Laboratory Genetic Metabolic Diseases, Amsterdam UMC, Emma Children’s Hospital, University of Amsterdam, Amsterdam, The Netherlands; 5 Amsterdam Gastroenterology Endocrinology Metabolism, Inborn Errors of Metabolism, Amsterdam, The Netherlands; 6 Core Facility Metabolomics, Amsterdam UMC location University of Amsterdam, Amsterdam, The Netherlands; 7 United for Metabolic Diseases, Amsterdam, The Netherlands; 8 Medicines for Society, Platform at Amsterdam UMC, University of Amsterdam, Amsterdam, The Netherlands

**Keywords:** Bile acid synthesis defects, Cholic acid, Toxic bile acid intermediates, Single enzyme defects, Zellweger spectrum disorders

## Abstract

**Background:**

Bile acid synthesis defects (BASDs) can be severely disabling involving the liver and nervous system, potentially due to elevated levels of toxic C_27_-bile acid intermediates. Cholic acid (CA) supplementation is hypothesized to decrease bile acid production, stimulate bile secretion and -flow, and slowing down disease progression. This systematic review assesses the clinical and biochemical effectiveness, and safety of CA in BASDs patients.

**Methods:**

A systematic review of MEDLINE, Embase and clinical trial registries (ClinicalTrials.gov, ICTRP registry) using controlled MeSH- and Emtree terms.

**Results:**

From 526 articles 70 publications were deemed eligible for inclusion based on title and abstract. 14 publications were included after full-text assessment comprising case reports and -series with 1–35 patients (162 patients in total) receiving 1 week to 16,5 years of CA treatment. All presented data on effectiveness, 8 studies also presented data on safety. The included population concerned patients with Zellweger spectrum disorders (*n* = 73), 3β-Hydroxy-Δ5-C_27_-steroid oxidoreductase deficiency (*n* = 62), cerebrotendinous xanthomatosis (*n* = 22), Δ4-3-oxosteroid 5β-reductase deficiency (*n* = 13), and α-methylacyl-CoA racemase deficiency (*n* = 3). Main outcomes concerned liver disease (12 studies), general physical examinations, biochemical outcomes, and safety (9 studies), and fat-soluble vitamin absorption (7 studies). The overall risk of bias score was considered to be critical (1 study), serious (4 studies), and moderate (9 studies). Major issues were missing data (10 studies), generalized data (8 studies), and no wash-out between treatments (4 studies).

**Conclusion:**

More controlled studies are required as the available data is insufficient to draw definite conclusions on the effectiveness and safety of CA treatment in BASD patients. Establishing an independent international disease registry could better utilize existing real-world data.

**Supplementary Information:**

The online version contains supplementary material available at 10.1186/s13023-024-03449-7.

## Background

Bile acid synthesis defects (BASDs) such as single enzyme deficiencies (SEDs) in bile acid synthesis or those resulting from generalized peroxisomal impairment in Zellweger spectrum disorders (ZSD) can lead to a variety of often severe disabling symptoms [1–4]. It is still largely unknown which biochemical abnormalities, either alone or in combination, contribute to the different clinical manifestations (e.g. growth retardation, neurological symptoms, liver dysfunction and ultimately liver failure). SEDs involve inherited deficiencies in enzymes responsible for catalysing key reactions in the synthesis of primary bile acids cholic acid (CA) and chenodeoxycholic acid (CDCA). Whereas involve defects in peroxisomal enzymes that are involved in the synthesis and transport of bile acids. Abnormally elevated bile acid intermediates, such as C_27_-bile acid intermediates dihydroxycholestanoic acid (DHCA) and trihydroxycholestanoic acid (THCA), are thought to be toxic and to contribute to the liver disease [1, 3–10]. However, a role for other biochemical abnormalities cannot be ruled out. Possibly, these intermediates are also toxic for the brain [11, 12]. Bile acid supplementation with CA for the treatment of different types of rare diseases caused by BASDs has been available for many years [13–16]. The hypothesis is that CA supplementation decreases bile acid production, stimulates bile secretion and improves bile flow and micellar solubilisation, thereby improving the biochemical profile and hopefully slows down the progression of the disease in patients with certain types of BASD [2, 5, 8, 17]. A number of studies show that CA treatment indeed reduces concentrations of toxic bile acid intermediates in plasma [9, 13, 18, 19]. In addition, several small clinical studies and observations have been published, which have led to authorization of cholic acid as an orphan drug in the EU for a selected number of SED indications: 3β-hydroxy-Δ^5^-C_27_-steroid oxidoreductase- (3β-HSD, HSD3B7) and Δ^4^-3-oxosteroid-5β-reductase (5β-reductase) deficiency [15]. The authorization for indications sterol 27-hydroxylase (cerebrotendinous xanthomatosis, CTX), α-methylacyl-CoA racemase (AMACR) deficiency and cholesterol 7α-hydroxylase (CYP7A1) deficiency was withdrawn in 2020 at the request of the marketing authorization holder due to commercial reasons [16]. In the United States CA is also authorized as a adjunctive treatment for peroxisomal disorders, including ZSD patients who exhibit manifestations of liver disease, steatorrhea or complications from decreased fat soluble vitamin absorption [20]. Despite the heterogeneity with respect to patient population, pathogeneses and clinical and biochemical presentations, the treatment is similar with a recommended dose of 10–15 mg/kg per day [21, 22]. 

To improve our understanding of the potential clinical and biochemical effectiveness of CA in patients with a SED or ZSD, we performed a systematic review of the literature. The objective of this systematic review was to evaluate the available data on effectiveness and safety of CA treatment in patients with the specified BASDs. Data was assessed through (1) determining the degree of suppression of bile acid synthesis and the improvement in clinical symptoms, and (2) reported side effects.

## Methods

This systematic review protocol is registered with PROSPERO (CRD42021214155). Standard systematic review methodology was used, aimed at minimizing bias, with reference to the Centre for Reviews and Dissemination (CRD) guidance for undertaking systematic reviews in health care [23]. 

### Search strategy

Relevant studies were identified by conducting searches in the following electronic databases: OVID MEDLINE, OVID Embase and clinical trial registries (ClinicalTrials.gov, ICTRP registry) using controlled MeSH terms (MEDLINE) and Emtree terms (Embase), and free text terms from the concepts: (1) cholic acid (or brand names including, but not limited to, Kolbam, Cholbam and Orphacol), and (2) the bile acid synthesis defects: 3-beta-hydroxydelta-5-C27-steroid oxidoreductase deficiency (3β-HSD), delta-4-3-oxosteroid-5-beta-reductase deficiency (5-beta-reductase, AKR1D1), sterol 27-hydroxylase deficiency (cerebrotendinous xanthomatosis, CTX), alpha-methylacyl-CoA racemase (AMACR) deficiency, cholesterol 7-alpha-hydroxylase (CYP7A1) deficiency, and Zellweger spectrum disorder (ZSD). Identified records were imported into Rayyan and duplicate records were removed. Reference lists and citing articles were crosschecked if deemed relevant. Details on the search strategies, as well as used search terms, are provided in Appendix I.

### Study selection criteria

**Fig. 1:**
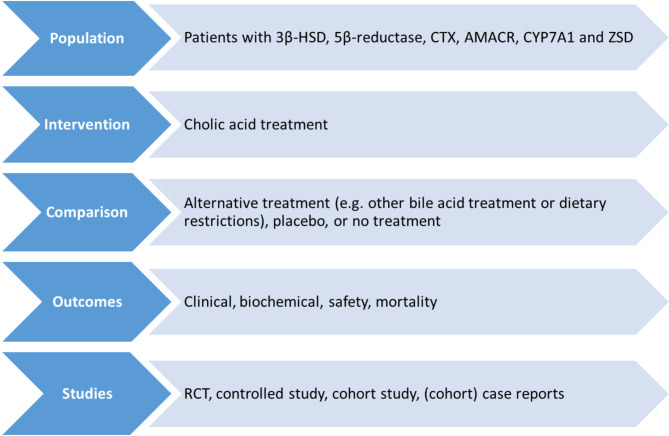
PICOS chart detailing inclusion criteria for systematic review. *Abbreviations* RCT, randomized controlled trial; PICOS, ‘population, interventions, comparators, outcomes, and study designs’. **Clinical outcomes**: Liver disease, kidney dysfunction, neurological disease, hearing impairment, vision impairment, skeletal disease or growth retardation, psychiatric symptoms. **Biochemical outcomes**: Primary bile acids and bile acid intermediates (toxic metabolite) plasma levels, liver chemistries, fat-soluble vitamins, plasma cholestanol and cholesterol levels. **Safety outcomes**: Dose-response relationship, adverse effects and side effects

Figure 1 shows the selection criteria that were used in conducting this systematic review. Our inclusion criteria included randomized controlled trials (RCTs), controlled studies, cohort studies, and case reports. No date restrictions were applied. Language was restricted to English and German. The search was restricted to studies with human subjects.

**Fig. 2:**
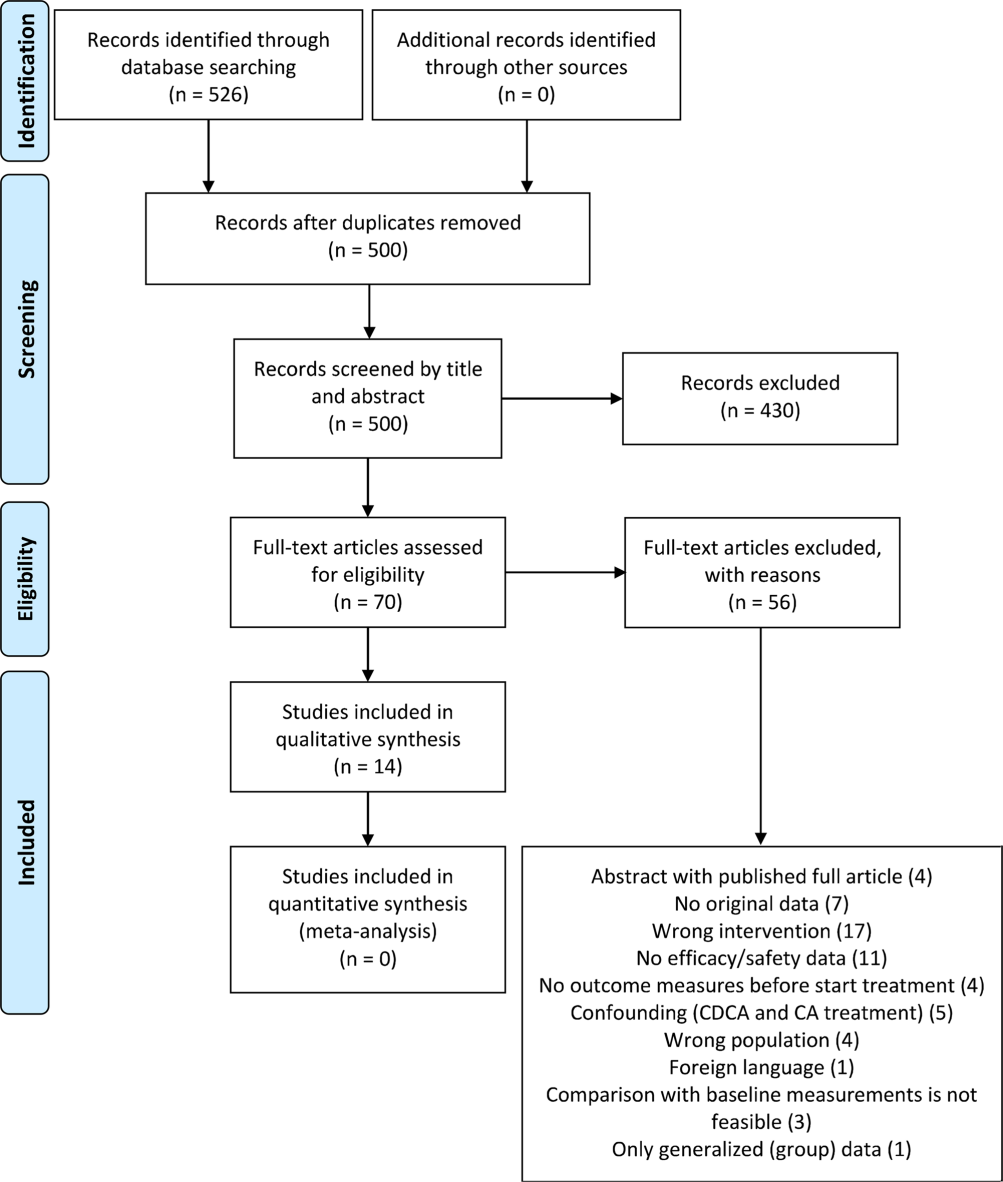
PRISMA flow diagram [25]

Two reviewers independently screened titles and abstracts of all articles retrieved to identify studies that potentially meet the inclusion criteria using Rayyan software tool [24]. Articles considered to be potentially relevant by both reviewers were retrieved and the full reports were assessed for eligibility according to the criteria (Fig. 1). Studies that did not meet all the criteria were excluded. Any discrepancies were resolved by consensus. Reasons for excluding studies were documented and summarized in the PRISMA flow diagram (Fig. 2) [25]. 

### Data extraction and assessment of bias

Data on the following outcomes were extracted:

1. Biochemical effects (change in plasma levels)):

- primary bile acids (CDCA and CA).
- toxic metabolites (THCA, DHCA).
- liver chemistries (e.g. ALT/AST, GGT, AF, bilirubin, PT, aPTT, factor V/VII).
- fat-soluble vitamins (A, D, E), cholestanol and cholesterol.

2. Clinical effects (occurrence, severity and change):

- liver disease (e.g. liver chemistries [ALT/AST, GGT, AF, bilirubin, PT, aPTT, factor V/VII)], hepatomegaly, fibrosis, cholestasis)
- kidney dysfunction (e.g. albumin-creatinine ratio, eGFR).
- neurological disease (e.g. cerebellar atrophy, leukodystrophy, cerebellar ataxia, peripheral/sensorimotor neuropathy, seizures, cognitive impairment).
- hearing impairment (e.g. sensorineural deafness).
- vision impairment (e.g. retinopathy and cataracts).
- skeletal disease and/or growth retardation (e.g. height/weight, osteoporosis).
- psychiatric symptoms (e.g. depression, manic episodes).

3. Safety (occurrence and severity):

- adverse effects.
- side effects.

Data were independently extracted by two reviewers using a standardized data extraction form in Castor EDC [26]. Outcomes of which no measurements were reported after initiation of treatment or for which no untreated baseline value was reported were not included in the data extraction. When possible, individual patient data were extracted, group effect data were not extracted unless individual patient data were given. Any discrepancies were resolved by consensus. When multiple publications of the same study were identified, data were extracted from the original publication or, in case additional data were given in a subsequent publication, reported as a single study.

The following data were extracted:

- PICOS items: patient population, intervention, comparator, outcome measures (see details below), and study design (Fig. 1).
- Study duration, dose, treatment duration, and number of subjects treated.
- Risk of bias (RoB) assessment based on the extracted data using appropriate RoB assessment tools (Murad or ROBINS-I tool) [27, 28].

### Data synthesis

Of the extracted data, differences between baseline and follow-up were synthesized. Due to the rarity and heterogeneity of the diseases of interest, and the heterogeneity of reported outcomes, it was not possible to perform a statistical- or meta-analysis of the data. Moreover, due to the heterogeneity of the data it was ultimately decided to first determine whether the extracted data could be marked as clinically or biochemically relevant according to specific criteria (Table 1). The data that have been deemed clinically or biochemically relevant were presented in this review in structured tables and as narrative summaries.

A narrative synthesis of the data was made through critical reading of the studies. Subgroup analyses were planned by type of BASD and outcome measure where possible, as we anticipated heterogeneous study designs and outcome measures. The data on clinical- and biochemical effects and safety of cholic acid treatment were synthesized through a narrative review with full tabulation of included articles.

**Table 1:** Criteria for changes in clinical and biochemical parameters to be marked as relevant

Clinical effect	Marked as positive change	Marked as negative change
• liver disease (e.g. hepatomegaly, fibrosis, cholestasis)	Improvement in Fibrosis score (F1-F4) score [29]	Deteriorating in score
• kidney dysfunction (e.g. albumin-creatinine ratio, eGFR)	Improvement in kidney disease grade (2–5) [30]	Deteriorating in grade
• neurological disease (e.g. cerebellar atrophy/leukodystrophy, cerebellar ataxia, peripheral/sensorimotor neuropathy, seizures, cognitive impairment)	Decreased severity and/or number of symptoms/episodes	Increased severity and/or number of symptoms/episodes
• hearing impairment (e.g. sensorineural deafness)	Improvement of hearing test results	Deteriorating hearing test results
• vision impairment (e.g. disturbed vision; eye movement disorders retinopathy and cataracts)	Improvement or stabilization of symptoms and/or eye test results	Deterioration of symptoms and/or eye test results
• skeletal disease and/or growth retardation (e.g. height/weight, osteoporosis)	Decreased severity and/or number of symptoms	Increased severity and/or number of symptoms
	Weight/ height SD score < -2 SD at baseline and increases with at least + 0.5 SD	Weight/height SD-score decreases with at least 0.5 SD to a value < -2 SD
• psychiatric symptoms (e.g. depression, manic episodes)	Decreased severity and/or number of symptoms/episodes	Increased severity and/or number of symptoms/episodes
**Biochemical effects**		
• primary bile acids (CDCA and CA) plasma levels	Value ≥ 2 x baseline value and baseline measurement ≤ 0.5 x LLN	Value ≤ 0.5 x baseline value and final value < LLN
• toxic metabolites (THCA and DHCA) plasma levels	Value ≤ 0.5 x baseline value and baseline measurement was at least > 2 x ULN	Value ≥ 2 x baseline value and final value > ULN
• liver chemistries (e.g. ALT/AST, GGT, AF, bilirubin, PT, aPTT, plasma levels)	Value ≤ 0.5 x baseline value and baseline measurement was ≥ 2 x ULN	Value > 2 x baseline value and final value > 2 x ULN
• fat-soluble vitamins (A, D, E), cholestanol and cholesterol plasma levels	Value ≤ 0.5 x baseline value and baseline measurement was ≥ 2 x ULN	Value ≥ 2 x baseline value and final value > ULN

## Results

The systematic search retrieved 526 articles (Fig. 2). Following the removal of duplicates, 500 unique publications remained, of which 430 publications were excluded based on title and abstract, leaving 70 publications deemed eligible for inclusion and these were assessed for full-text screening.

Ultimately, 14 publications were included for qualitative analysis after full-text screening. Main reasons for exclusion were wrong intervention (17 studies), no data on effectiveness or safety (11 studies), or no original data (7 studies) (Fig. 2). The reasons for exclusion after full texts had been viewed are presented in Supplementary Table 2. The included publications comprised case reports and case series with 1–35 patients (per disease type). Ultimately these studies included a total of 162 patients, receiving CA monotherapy as intervention. The studies lasted 1 week up to 16,5 years. Most publications presented data on both safety and effectiveness (*n* = 9). The majority of the people included in the studies concerned patients with ZSD (*n* = 73), followed by patients with a 3β-HSD defect (*n* = 62), CTX patients (*n* = 22), AKR1D1 patients (*n* = 13), and AMACR patients (*n* = 3). No publications were found studying the safety or effectiveness of CA in patients with a CYP7A1 defect. Of the 14 included publications, four studies included two or more types of bile acid synthesis defects, of which 1 publication had a population of all five diseases. Full details of the conduct and characteristics of each included publication can be found in Table 2.

**Table 2:** Summary of results and characteristics of **included** studies

Study			Treatment				Data		Population (*n* patients)						Outcomes			
First Author	Year	Study type	Intervention	Comperator	Treatment Duration	Number of patients	Safety data	Effectiveness data	CTX	3β-HSD	AKR1D1	CYP7A1	AMACR	ZSD	Primary outcomes	Safety reported (safety population *n*)	AEs reported (*n*)	AE total of patients (*n*)
Ahmad [31]	2019	Case series	CA 10 mg/kg	-	16 months − 3 years	4		X		X (4)					-Liver function -Fat-soluble vitamin levels -Biochemical response (serum)	No	.	.
Berendse [18]	2016	Case series	CA 10–20 mg/kg	-	9 months	19	X	X						X (19)	-Degree of suppression of bile acid synthesis - The change in plasma C24-bile acid levels	Yes (19)	Yes (4)	4
Al-Hussaini [32]	2017	Case series	CA 15 mg/kg	-	1 month − 8 years	15	X	X		X (11)	X (3)			X (1)	-Liver function -Fat-soluble vitamin levels -Biochemical response (urine)	Yes (15)	Yes (1)	1
Klouwer [19]	2019	Case series	CA 10–20 mg/kg	-	3–21 months	22^Δ^	X	X						X (22)	-Degree of suppression of bile acid synthesis - Presence of FGF19	Yes (22)	Yes (6)	6
Bossi [33]	2017	Case report	CA 12 mg/kg	-	2 years	1	X	X		X (1)					-Weight/ height -Liver function (histology) -Biochemical response (urine)	Yes (1)	.	.
Duran [34]	2013	Case report	CA ?	-	Unknown	1		X		X (1)					-Liver function	No	.	.
Gonzales [14]	2009	Case series	CA 6.3–14.2 mg/kg	-	5 years	15	X	X		X (7)*	X (0)*				- Liver biochemistry - Biochemical response (urine)	Yes (15)	Yes (5)	4
Heubi [35]	2017	Case series	CA 10–15 mg/kg	-	Unknown	82	X	X	X (5)	X (35)	X (10)		X (1)	X (31)	- Changes from pre- to post-treatment in atypical urinary bile acids and liver chemistries - Height and weight	Yes (79)	Yes (114)	38
Heubi [36]	2018	Case report	CA 9-18.8 mg/kg	-	17.5 years		X	X						X (1)	- Change in liver chemistries - Change in bile acids (urine)	Yes (1)	Yes (1)	1
Koopman [13]	1985	Case series	CA 750 mg/day	-	20–315 days	5	X	X	X (5)						- Change in urinary excretion of 5ß-cholestane-3α,7α,12α,23,25-pentol	Yes (3)	.	.
Mandia [37]	2019	Case series	CA 500–750 mg/day	-	71-1311 days	12	X	X	X (12)						- Clinical status - Cholestanol levels - Adverse effects	Yes (12)	Yes (1)	1
Rogers [38]	2018	Case report	CA 10 mg/kg	-	1 week	1		X		X (1)					- Change in liver chemistries - Change in cholestasis	No	.	.
Setchell [39]	2003	Case report	CA 15 mg/kg	-	7 years	1		X					X (1)		- Change in bile acid levels (urine, bile, serum and feces)	No	.	.
Woollett [40]	2006	Case series	CA ? mg/kg	No treatment	3 weeks	3		X		X (2)			X (1)		- Cholesterol absorption and fractional syntetic rates	No	.	.

Abbreviations and symbols: FGF19, Fibroblast growth factor 19; ?, information unknown

*Five 3β-HSD patients and one AKR1D1 patient were excluded from this review because they had received previous treatment with ursodeoxycholic acid (UDCA) with no clear wash-out period and pre-CA treatment measurements

Δ19 patients also participated in the study by Berendse et al., and received continuation of CA treatment

The majority of the included studies described biochemical outcomes (*n* = 9), liver disease outcomes (including liver histology and liver elasticity, *n* = 12), and the absorption of fat-soluble vitamins (*n* = 7) (Table 3). Ten studies looked at clinical outcomes such as general physical examination (*n* = 9), neurological disease (*n* = 2), vision impairment (*n* = 1) and psychiatric symptoms (*n* = 1). Two studies reported data on kidney dysfunction. A total of nine studies recorded safety parameters. Four studies reported data on mortality. No studies reported data on hearing impairment or skeletal disease.

**Table 3:** Summary of reported outcomes

Outcome	Studies describing outcome		Studies describing relevant improvement		Studies describing relevant deterioration		Studies describing no relevant change		Studies excluded from data synthesis		
	No.	Ref	No.	Ref	No.	Ref	No.	Ref	No washout	No pre- and post- treatment data	Generalized (aggregated data / surrogate endpoint^1^)
Physical examination (general)	9	[14, 18, 19, 31–33, 35, 39, 40]	2	[14, 19]	1	[19]	3	[14, 19, 39]	-	[32–35, 37, 38]	[18, 31, 32, 35,40]
Biochemical response	9	[13, 14, 18, 19, 31–33, 35, 39, 40]	2	[18, 19]	2	[18, 19]	2	[18, 19]	[13]	[32, 33, 39]	[13, 14, 32, 35, 40]
Fat-soluble vitamins	7	[18, 19, 31–33, 38, 39]	1	[31]	-	-	-	-	-	[18, 19, 32, 33, 38, 40]	[18, 19, 32]
Liver disease	12	[14, 18, 19, 31–35, 37–40]	6	[19, 31, 34, 38, 30]	2	[19, 40]	3	[19, 31, 39]	[14, 33, 37]	[14, 18, 32, 37]	[18, 32, 35]
Kidney dysfunction	2	[14, 33]	1	[33]	-	-	-	-	[14]	-	-
Neurological disease	2	[37, 39]	1	[37]	1	[37]	-	-	-	[39]	-
Hearing impairment	0	-	-	-	-	-	-	-	-	-	-
Vision impairment	1	[37]	-	-	-	-	-	-	-	[37]	-
Skeletal disease/growth retardation	0	-	-	-	-	-	-	-	-	-	-
Psychiatric symptoms	1	[37]	-	-	-	-	-	-	-	[37]	-
Mortality	4	[18, 19, 32, 35]	-	-	-	-	-	-	-	-	-

1 Surrogate endpoints concern measurements that are not itself a direct measure of clinical benefit, but are thought to be reasonably likely to predict the clinical benefit

**Table 4:** Summary of relevant biochemically data

Biochemical marker	Abnormal pre-treatment value (*n*/total *n*)	Improvement (*n*)	Deterioration (*n*)	Stable (*n*)
CA	Abnormal* (0/19)	-	-	-
	Normal (19/19)	NA	0	19
DHCA	Abnormal^±^ (17/22)	13	0	4
	Normal (5/22)	NA	3	2
THCA	Abnormal^±^ (15/22)	12	1	2
	Normal (7/22)	NA	0	7

Not applicable (NA), baseline value <0.5 x LLN (*), baseline value >2 x ULN (±). CA reference value 0.1 - 4.7 μmol/L; DHCA reference value <0.05; THCA reference value <0.05 - 0.1 μmol/L

### Clinical and biochemical relevancy of included data

Most of the included studies reported aggregated data, presenting overall group results, not always differentiating for the disease type when the patient group was heterogeneous. In many cases pre- or post-treatment measurements were missing from the reports, or no numerical (individual) data was reported, but the findings were reported in a descriptive manner, such as “a marked reduction in the atypical bile acids” or “symptoms normalized” without definition. There were also a number of patients that received another bile acid therapy (i.e. ursodeoxycholic acid (UDCA) or CDCA) before receiving CA treatment. In these instances it was not always clearly defined whether there was a wash-out period between the treatments. The reported pre-treatment outcome measurements were often from before the first bile acid treatment, or it was unclear at which moment samples were collected. In these cases it was not possible to interpret the effect of CA treatment based on the results, and these data have therefore been excluded from the data presentation in this review (Table 3). The outcome measurements for which interpretable pre- and post-treatment outcome measurements had been reported are summarized per category. Ultimately individual patient data have been summarized in the Supplementary Tables.

### Biochemical response

Nine studies were found reporting data on biochemical response of CA, of which the data of only two studies could be tested for relevance according to our criteria (Table 1). The study by Klouwer et al. was a continuation of the study by Berendse et al. [18, 19]. Hence, the patient population was the same, except for three additional participants in the study by Klouwer et al. Patients were treated with CA for 9 months in the study by Berendse et al. and the treatment was continued for an additional 12 months in the study by Klouwer et al. The results on biochemical outcome measurements (CA, DHCA, THCA) have been summarized in Table 4. The complete individual data of the reported CA, DHCA and THCA plasma levels can be found in Supplementary Table 3.

#### Cholic acid

In the study by Berendse et al. there were no patients with abnormally low pre-treatment CA plasma levels (< 0.5 x LLN) [18]. Two patients had strongly elevated CA levels (> 2 x ULN) before CA treatment started (11.0 and 47.4 µmol/L). CA levels increased in all but one patient, but none of these changes could be marked as clinically relevant as values were never below the reference range (0.1–4.7 µmol/L). Post-treatment values were higher than the upper reference range in nine patients of whom the CA levels further strongly increased during CA treatment (154.2 and 251.8 µmol/L respectively) (Supplementary Table 3).

#### Bile acid intermediates (DHCA/THCA)

In the study by Klouwer et al. (extended study from Berendse et al.) 16/22 patients had increased pre-treatment DHCA plasma levels (> 2x ULN) and THCA levels were increased in 15/22 [19]. DHCA and THCA plasma levels improved in 13/16 and 12/15 patients respectively. In most patients with improved DHCA plasma levels, the THCA levels had also improved (*n* = 11). Of the 22 patients seven patients reached improved plasma levels < 0.05 µmol/L for DCHA and/or THCA after 21 months CA treatment. In three patients the DHCA plasma levels had deteriorated after 21 months of treatment, however the DHCA value could still be considered relatively low (0.1 µmol/L). In one patient the THCA plasma levels had deteriorated (i.e. increased from 10.6 to 16.7 µmol/L), however this patient had dropped out of the study by Berendse et al. after 3 months of CA treatment due to persistently elevated conjugated bilirubin levels after dose reduction, but was still monitored in the study by Klouwer et al. Of the ‘stable’ patients only one patient had seriously elevated (> 1 µmol/L) DHCA and THCA levels at baseline (15.5 and 12 µmol/L respectively). The DHCA and THCA plasma levels remained extremely elevated after 21 months of CA treatment (10.5 and 11.5 µmol/L respectively)(Supplementary Table 3).

#### Fat-soluble vitamin absorption

Seven studies were found reporting data on fat-soluble vitamin absorption, of which the data of only one study could be compared to the criteria mentioned in Table 1. This study by Ahmad et al. reported data on the change in 25-hydroxy vitamin D (25-OH Vitamin D) plasma levels for two of four 3β-HSD patients who were treated with CA 10 mg/kg/day in this case series [30]. Both patients had persistent low vitamin D deficiency pre-treatment, with 25-OH Vitamin D levels below 4 ng/mL despite receiving vitamin D supplementation of 50.000 IU per day. After 20 months of CA treatment both patients had a significant increase in 25-OH Vitamin D with plasma levels of 41 ng/mL (reference value > 20 ng/mL). The levels of 1,25 dihydroxy vitamin D remained unchanged during treatment and were within reference range (25 — 86,5 pg/mL) for both patients. According to the study, the patients did not receive, nor required, any vitamin supplements while on CA treatment.

### Clinical effects – liver disease

Twelve studies were found reporting data on liver disease, presenting data on either physical examination of cholestatic liver disease, liver chemistries, liver elasticity and/or liver histology. Of these twelve studies, the reported data of seven studies could be compared to the criteria mentioned in Table 1. Data on liver chemistries AST, ALT, conjugated bilirubin, AF, PT and GGT were presented in these seven included studies. Relevant individual patient data was collected from six studies for AST, ALT, AF, conjugated bilirubin, PT and GGT. Three studies presented data on liver elasticity (i.e. Fibroscore/METAVIR score) and one study presented data on liver histology [14, 18, 19, 29]. 

#### Physical examination of cholestatic liver disease

Gonzales et al. reported on the presence and development of jaundice, hepatomegaly, fatty stools and areflexia (Table 5) [14]. All patients showed at least one of these symptoms at the start of CA treatment (*n* = 7). Patient individual data on treatment dose or duration were not reported. Initial mean daily dose was 14.2 mg/kg, after 5 years of treatment mean daily dose had decreased to 6.3 mg/kg (range 3–9 mg/kg). Following 5 years CA treatment, the symptoms disappeared in most patients (*n* = 5). In two patients hepatomegaly remained during and after 5 years CA treatment. As no further specifications on these symptoms were given for the individual patients, the data could only be interpreted as a symptom being present or absent.

**Table 5:** Summary of data on clinically relevant general physical condition, individual patient data

Study	Disease	CA dose (mg/kg/day)*	Treatment duration	Jaundice		Hepatomegaly		Fatty stools		Areflexia	
				Pre-treatment	Post-treatment	Pre-treatment	Post-treatment	Pre-treatment	Post-treatment	Pre-treatment	Post-treatment
Gonzales et al.	3β-HSD	NA	NA	-	-	**+**	-	**+**	-	-	-
	3β-HSD	NA	NA	+	-	**+**	+	**+**	-	**+**	-
	3β-HSD	NA	NA	-	-	**+**	-	**-**	-	**+**	-
	3β-HSD	NA	NA	-	-	**+**	-	**-**	-	**+**	-
	3β-HSD	NA	NA	+	-	**+**	+	**+**	-	-	-
	3β-HSD	NA	NA	+	-	**+**	-	**+**	-	-	-
	3β-HSD	NA	NA	-	-	**+**	-	**+**	-	-	-

Symptom present (+), symptom absent (-), data not available (NA)

**Table 6:** Summary of relevant data on liver chemistries

Liver parameter	Abnormal pre-treatment value (*n*/total *n*)	Improvement (*n*)	Deterioration (*n*)	Stable (*n*)
AST	Abnormal^±^ (4/24)	2	0	2
	Normal (20/24)	NA	0	24
ALT	Abnormal^±^ (4/30)	3	0	1
	Normal (26/30)	NA	2	24
Conjugated bilirubin	Abnormal^±^ (2/20)	0	0	2
	Normal (18/20)	NA	2	16
AF	Abnormal^±^ (4/4)	3	0	1
	Normal (0/4)	NA	NA	NA
PT	Abnormal^±^ (1/1)	1	0	0
	Normal (0/1)	NA	0	0
GGT	Abnormal^±^ (1/1)	1	0	0
	Normal (0/1)	NA	0	0

± Baseline value >2 x ULN. Improvement: post-treatment value ≤0.5 x baseline value and baseline measurement was ≥2x ULN; deterioration: post-treatment value > 2x baseline value and post-treatment value > 2 x ULN. Reference values: ALT (alanine transaminase), <40 U/L; AST (aspartate aminotransferase), <45 U/L;AF (alkaline phosphatase), <123 U/L; conjugated bilirubin, <5 μmol/L; PT (prothrombin time), 9.7 - 11.9 seconds; GGT (gamma-glutamyl transferase), <62 U/L. Not applicable (NA)

**Table 7:** Summary of relevant data on fibrosis score

Parameter	Abnormal pre-treatment value (*n*/total *n*)	Improvement (*n*)	Deterioration (*n*)	Stable (*n*)
Fibrosis score	Abnormal^±^ (14/22)	6	1	7
	Normal (12/22)	NA	0	12

± Fibrosis score ≥ F2 (< 7.1 kPa (F1); ≥ 7.1 kPa - < 9.5 kPa (F2); ≥ 9.5 kPa - < 12.5 kPa (F3); ≥ 12.5 kPa (F4) (41)). Not applicable (NA)

#### Liver chemistries

Seven studies presented clinically relevant data on the effect of CA treatment on liver chemistries [18, 19, 29–33]. In Table 6 a summary is presented of the relevant available individual patient data . The majority of the patients that participated in the studies had normal AST, ALT and conjugated bilirubin levels at the start of CA treatment and most of these patients remained stable during CA treatment (Table 6). Pre-treatment AST values were increased (≥ 2 x ULN) in four patients, of which two patients had an improved (0.5 x pre-treatment value) and normalized AST value after CA treatment. The other two patients were considered to be stable for AST levels, yet these remained elevated. No patients with normal or elevated AST plasma levels at the start of CA treatment showed deterioration in this outcome. Pre-treatment ALT values were increased (≥ 2 x ULN) in 4 patients and normal in 26 patients. Three patients with increased pre-treatment ALT values showed an improvement (0.5 x pre-treatment value) for this outcome after CA treatment, the fourth patient was considered to be stable. Of the 26 patients with normal ALT pre-treatment values two patients showed deteriorated (2 x pre-treatment value and ≥ 2 x ULN), whereas the remaining 24 patients were considered to be stable.

Most patients had normal pre-treatment conjugated bilirubin levels (≤ 5 µmol/L) and remained stable throughout the treatment period. Specifically, of the 18 patients with normal pre-treatment levels, 16 remained stable, while two showed deterioration. Among the two patients with elevated pre-treatment conjugated bilirubin levels (> 2 x ULN), both remained stable post-treatment.

Individual data on AF, PT and GGT were only presented from a limited amount of patients (*n* = 4, 1 and 1 respectively). For these parameters all patients had elevated pre-treatment values of at least 2 x ULN (*n* = 4, *n* = 1 and *n* = 1 respectively). All patients showed improved post-treatment values (at least 0.5 x pre-treatment value), except for one patient who was considered to be stable (Table 6).

The complete individual data on the individual liver chemistry measurements can be found in Supplementary Table 4a and 4b.

#### Liver elasticity

Three studies report measurements on the liver elasticity of ZSD patients (*n* = 22) [18, 19] and 3β-HSD patients (*n* = 6) [14]. For the patients in the study by Berendse et al. and its continuation study by Klouwer et al. liver fibrosis was monitored over a total CA treatment period of 21 months using a non-invasive transient elastography (FibroScan^®^) analysis method [18, 19]. Patient individual data was given on liver elasticity in kPa. These numbers have been translated into fibrosis scores for ease of interpretation and comparison [34]. Two patients were ultimately excluded from the study because of persistent rise in levels of conjugated bilirubin and/or plasma transaminases [19]. The individual patient data has been summarized in Supplementary Table 5. As the study by Klouwer et al. is a continuation of the study by Berendse et al., only the post-treatment measurements of the study by Klouwer et al. were evaluated to determine whether the fibrosis score improved, deteriorated or remained stable with CA treatment (Table 7).

**Table 8:** Summary of relevant general physical condition (weight)

Outcome	Abnormal pre-treatment value (*n*/total *n*)	Improvement (*n*)	Deterioration (*n*)	Stable (*n*)
Weight SD score	Abnormal* (5/23)	3	1	2
	Normal (17/23)	NA	1	16

Not applicable (NA), Standard deviation (SD), pre-treatment weight SD value at least ± 2 SD (*), improvement: baseline ≤ -2.0 SD score and post-treatment value +0.5 SD or more compared to baseline, deterioration: post-treatment value ≤ -2,0 SD score and -0.5 SD or more compared to baseline, otherwise considered clinically stable

In the study by Gonzales et al. liver elasticity was determined on the basis of liver biopsy specimens collected before the start of CA treatment and after 5 years of CA treatment. A differentiation was made between portal fibrosis and activity, which were scored according to the METAVIR grading system, and lobular fibrosis, which was scored on a scale of 0 to 2 — with 0 indicating absence of fibrosis, 1 indicating moderate fibrosis, and 2 indicating marked fibrosis [14]. A total of 12 3β-HSD patients were followed in this study. However, the data of six 3β-HSD patients were excluded from this review as these patients were previously treated with UDCA and data on liver elasticity prior to CA-monotherapy was not available. The individual patient data on METAVIR fibrosis score of the six remaining patients has been summarized in Supplementary Table 6.

Ultimately for a total of 25 patients pre-treatment and post-treatment elasticity measurements have been reported, of whom the individual data were included for review. Of these patients, 14 patients had abnormal elasticity measurements, i.e. increased fibrosis scores (≥ F2) at the start of CA treatment (Table 7). After receiving CA treatment 6 patients showed an improvement in fibrosis score [14, 19]. One patient deteriorated in fibrosis score, The remainder of the patients (*n* = 19) were considered to be stable as the fibrosis stage didn’t change, or their fibrosis score remained ≤ F1 (Supplementary Tables 5 and 6) [14, 19]. 

#### Liver histology

Setchell et al. reported on the liver histology in a 10 week old AMACR patient. A liver biopsy showed giant cell transformation (GCT) and scattered necrotic eosinophilic hepatocytes (many derivatives of GCT). Intralobular cholestasis was described as moderate. After almost 8 months of CA treatment (15 mg/kg/day) a follow-up liver biopsy was performed. The biopsy revealed ‘minimal hepatocellular cholestasis’, ‘marked reduction’ in GCT and no stainable iron compared with the pre-treatment biopsy and there was no fibrosis [29].

### Clinical effects – kidney disease

One study reported clinically relevant data on kidney disease. In this study by Bossi et al. one 3β-HSD patient is presented with multiple micro cysts. The renal micro cysts had disappeared after two years of CA treatment [35]. 

### Clinical effects – neurological disease

Two studies, with a combined total of 12 CTX patients and 1 AMACR patient, reported data on neurological symptoms [29, 36]. One of these studies (Mandia et al.) reported individual pre- and post-treatment data on cognitive impairment and walking disorders for CTX patients (*n* = 12). However, the presented data is heterogeneous and for an important part subjective as the development of the symptoms were self-reported by caregivers as being present or not and whether or not an improvement, worsening or stabilization was seen after CA treatment. Therefore no objective interpretation of these data can be made [36]. The individual patient data this study is summarized in Supplementary Tables 7 to illustrate this.

### Clinical effects – vision impairment

Mandia et al. was the only study reporting on vision impairment [36]. The presence and development of cataracts was determined in 12 CTX patients. Almost all patients presented with cataracts in this study (*n* = 11) of which most patients required cataracts surgery before CA treatment was started and were therefore not considered for evaluation in the study (*n* = 7). Of the patients who didn’t receive cataract surgery the cataracts was reported to be stable in three. In one the evolution is unknown as the severity of the cataract was not objectified and its presence or absence at the start of CA treatment was unclear it is not possible to evaluate the effect of CA treatment on cataract.

### Clinical effects – growth retardation (weight SD score)

Nine studies reported data on general physical condition of the patients, of which three studies reported individual patient data [14, 19, 29] (Table 3), which could be assessed for clinical relevancy according to our criteria (Table 1). Klouwer et al. reported data on the weight of 22 ZSD patients after 3 to 21 months of CA treatment [19]. Setchell et al. reported data on the weight of one AMACR patient after 7 years of CA treatment [29]. A total of 23 patients were followed in the two studies, of which five ZSD patients were severely underweight (standard deviation (SD) scores (≤ -2 SD) and one ZSD patient was severely overweight ( ≥ + 2 SD). Of the five patients with severe underweight, three patients had an improved SD score ( ≥ + 0.5 SD) after 21 months of CA treatment, one ZSD patient had a deteriorated SD score, whereas for the other two patients the SD scores remained aberrant, yet stable (Table 8). One patient was increasingly underweight during CA treatment, with a pre-treatment SD score of -1.86 and a post-treatment SD score of -2.86. The patient individual data is presented in Supplementary Table 2.

### Clinical effects – psychiatric symptoms

One study from Mandia et al. reported on psychiatric symptoms in CTX patients, namely on presence of behavioral disorders [36]. No objective interpretation of these data can be made as it is self-reported data by caregivers on whether or not behavioral disorders were present or not, and whether or not an improvement, worsening or stabilization was seen after 71 to 1311 days of CA treatment.

### Safety

#### Adverse events

**Fig. 3:**
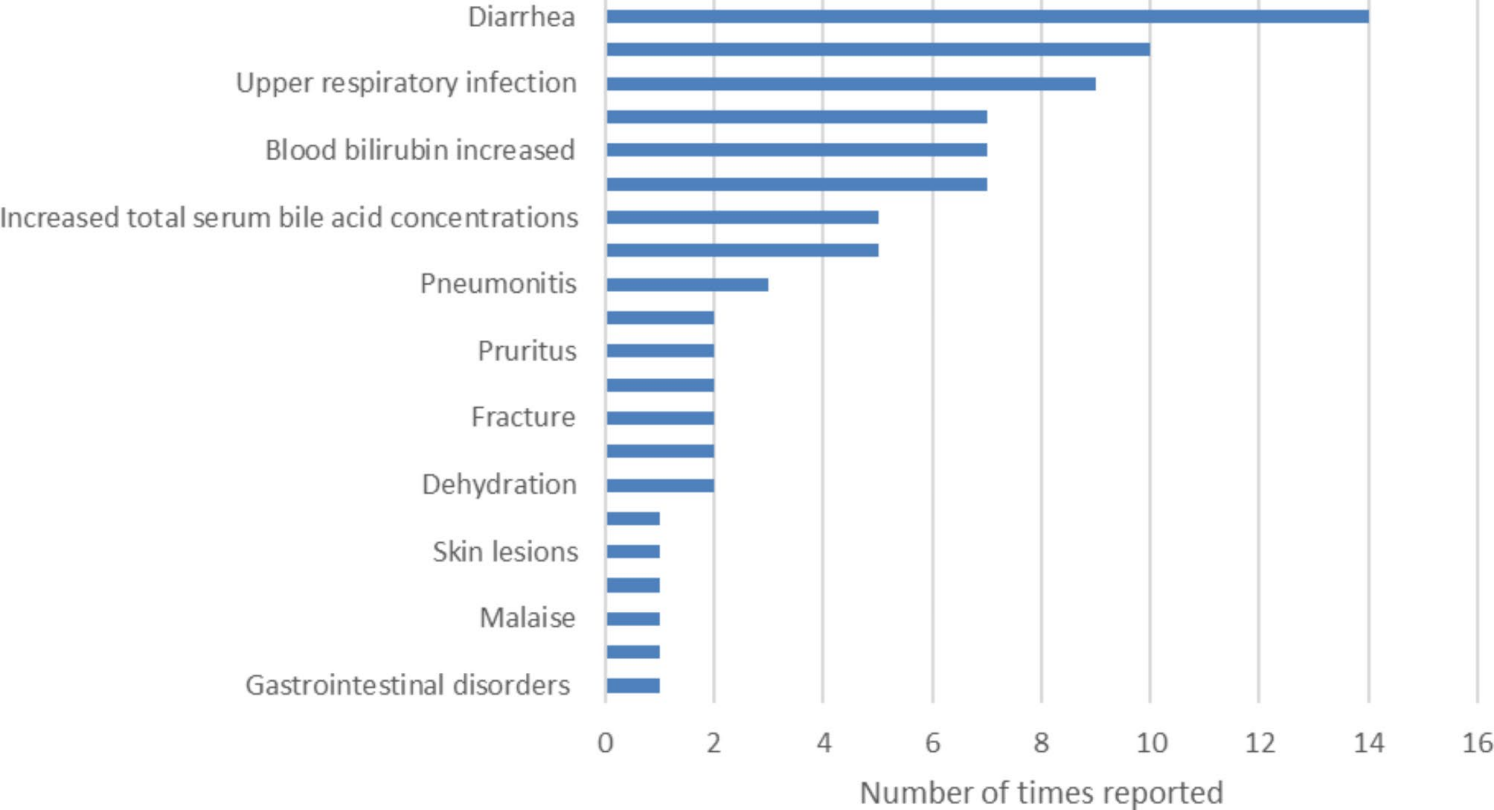
Types of (serious) adverse events and number of times reported

Adverse events (AEs) and serious adverse events (SAEs) were reported in 74 of 160 patients. A total number of 81 (S)AEs were reported in 7 studies (Fig. 3) [14, 18, 19, 36–39]. Mortality is discussed separately. Some SAEs can be categorized as disease progression or complications of the underlying condition (despite CA treatment): disease progression (*n* = 7), hepatic failure (*n* = 7) and hepatocellular carcinoma (*n* = 11) were reported in 15 patients. Other SAEs are more difficult to categorize (disease complication vs. side effect of CA) and included diarrhea (*n* = 9 patients), increased bilirubin blood levels (*n* = 7 patients), followed by pruritus and dehydration (both *n* = 2 patients) and malaise, skin lesions and urinary tract infection (all *n* = 1 patient) (Fig. 3). Disease progression and/or hepatic failure, and hepatocellular carcinoma (HCC) was reported for 14 and 1 patients respectively.

Gonzales et al. reported on cases of suspected (accidental) CA overdose in four children (56 mg/kg as a single dose). Increased serum GGT and ALT levels (associated with increased serum bile acid concentrations) were described signs of CA overdose. Serum GGT and ALT levels returned back to normal after CA dose was decreased. For one patient pruritus, diarrhea, as well as elevated serum GGT, ALT and total bile concentration (50 µmol/L) were signs of an accidental overdose [14]. 

#### Pregnancy

Gonzales et al. reported four uneventful pregnancies by two 3β-HSD patients during CA treatment [14]. 

### Mortality

Four studies with a combined total of 138 patients (ZSD, 3β-HSD, AKR1D1, CTX and AMACR) reported data on mortality (Table 9). In these studies death was reported for 24 patients [18, 19, 37, 39]. The cause of death was related to the underlying condition in the majority of patients (*n* = 13). For 9 patients the specific cause of death was unknown or not reported.

**Table 9:** Data on reported mortality per study and disease type

Study	Disease	*N* patients	Patient characteristics	Cause	Additional information
Heubi [35]	AKR1D1	4	AKR1D1 patients	Disease progression	End-stage liver disease at time of diagnosis and initiation of treatment, liver disease deteriorated despite CA therapy
Al-Hussaini [32]	AKR1D1	2	Female, presentation at 1 month old, neonatal cholestasis. CA treatment started at 3 months old, deceased at 4 months old	Liver failure	Liver failure was present at the time CA therapy was initiated, had also developed dilated cardiomyopathy and needed treatment for congestive heart failure.
			Male, presentation at 1,5 months old, neonatal cholestasis. CA treatment started at 2 months old, deceased at 6 months old	Heart failure (dilated cardiomyopathy)	Needed treatment for congestive heart failure, passed away “despite dramatic response to CA”
Berendse (18)	ZSD	0	-	-	-
Al-Hussaini [32]	ZSD	1	Female, presentation at 2 months old, neonatal cholestasis. CA treatment started at 3,5 months old, deceased at 3 years old	Complications related to this multiorgan primary disease	-
Klouwer [19]	ZSD	0	-	-	-
Heubi [35]	ZSD	15	Unknown	Unkown cause (*n* = 8)	-
				Worsening of the underlying condition (*n* = 4)	-
				Complications due to end-stage liver disease (*n* = 1)	-
				Seizures/cyanosis (*n* = 1)	-
				Not reported (*n* = 1)	Died before receiving CA treatment
Al-Hussaini [32]	3β-HSD	1	Male, presentation at 3 months old, neonatal cholestasis. CA treatment started at 6 months old, deceased at 9 months old	Liver failure	Liver failure was present at the time CA therapy was initiated, “failed to respond to CA therapy”.
Heubi [35]	3β-HSD	1	Unknown	Disease progression	-

### Quality assessment

**Fig. 4:**
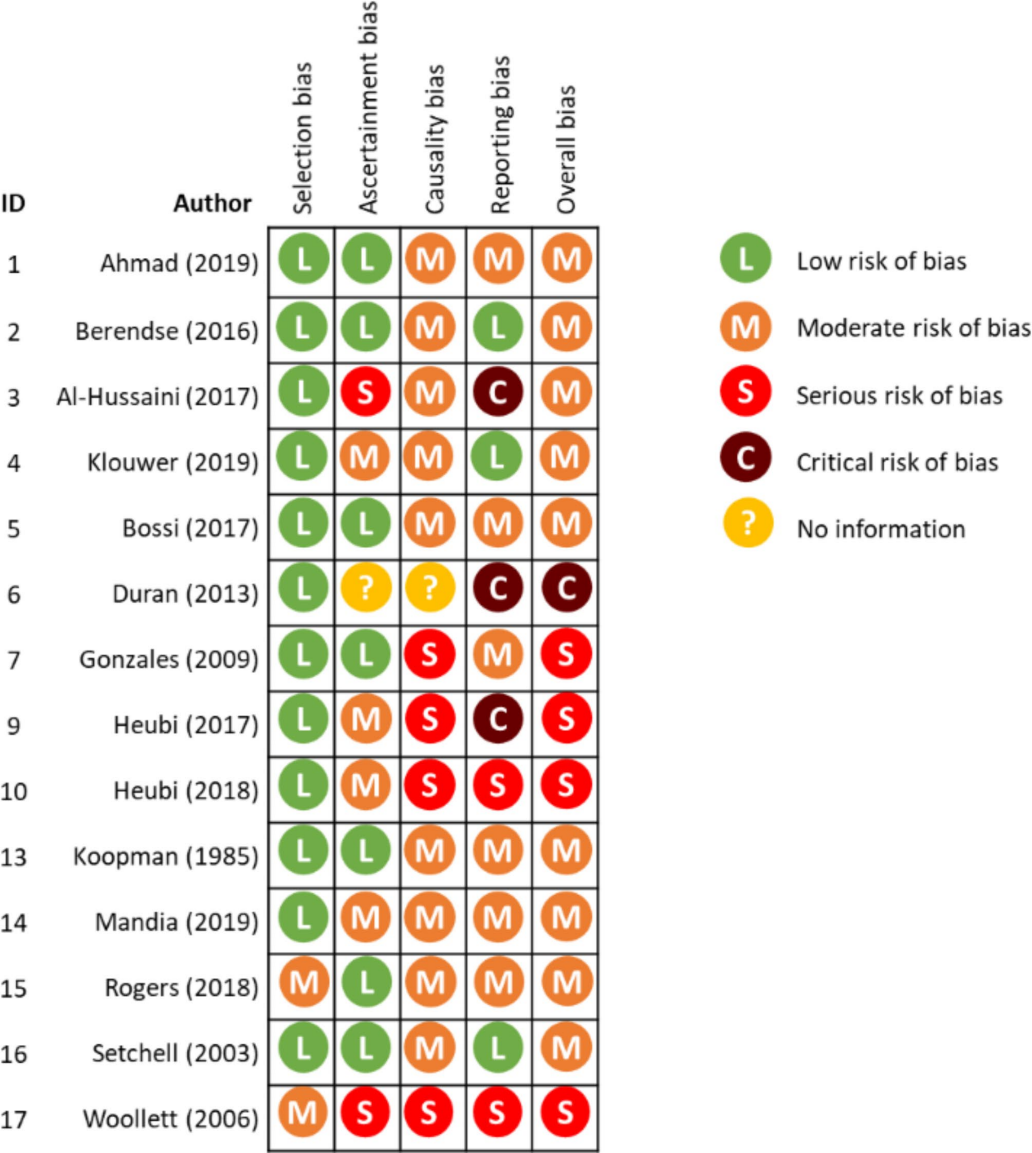
Risk of bias assessment of included studies using Murad tool. This represents the review authors judgements on each risk of bias item

A study quality assessment was performed for the fourteen included studies according to the Murads tool for risk of bias (RoB) assessment for case series and case reports [27]. The overall risk of bias score was considered to be critical for one study [31], serious for four studies [14, 33, 39, 40], and moderate for the remaining nine studies [13, 18, 19, 29, 30, 32, 35–37]. Bias in causality and reporting were the main issues of concern in the assessment (Fig. 4).

## Discussion

The objective of this systematic review was to evaluate the available data on effectiveness and safety of CA treatment in patients with the specified BASDs. Data were assessed through (1) determining the degree of suppression of bile acid synthesis and the improvement in clinical symptoms, and (2) reported side effects. A total of 162 patients were followed in the included 14 studies, this number may be lower given the study overlap that might include a patient more than twice [14, 18, 19, 38–42]. Overall, the studies had severe limitations that seriously jeopardize the interpretation of effectiveness. While we acknowledge the difficulties related to performing robust trials in rare diseases, specifically in those with a slowly progressive course, it is surprising that despite the many shortcomings, conclusions are frequently very positive about the effectiveness of the intervention. In this context, it should be noted that eight of the fourteen included studies involved co-authors with equity interests or expert roles in one of the two authorization holders of Orphacol or Cholbam [14, 30, 31, 33, 36–39]. In more detail: all studies had a limited number of included patients, and were unblinded and non-comparative. Only two studies were performed in a controlled setting, while the other 12 were mostly uncontrolled and observational. None of the studies examined clinically relevant outcome measures, which are also difficult to determine for some of the indications or require a long follow-up. The overall study duration was short; only four studies had a study period of 5 years or longer [14, 29, 37, 40]. In particular ZSD, CTX and AMACR deficiency are slowly progressive conditions in which the clinical presentation can remain the same for decades. In 13 studies data were either missing or were presented in a generalized group data. In two studies no differentiation was made between disease type when presenting group data [36, 42]. Ten studies reported outcome measurements in a descriptive manner for at least one outcome, claiming ‘improved’ or ‘worsened’ conditions without definition [14, 18, 19, 29, 31, 32, 35–37, 39]. One study presented a worst-to-best analysis, comparing the worst measurement with the best measurement that was found at any given time during the study for AST and ALT measurements [39]. Prior treatment with UDCA, CDCA or taurocholic acid (TCA) was a present confounder in four studies, without a clear wash-out period between the different treatments and clear baseline values for outcome parameters [13, 14, 35, 36]. Use of concomitant medication (i.e. vitamin supplements) was not always clearly reported in the studies. Moreover, in two studies CA treatment dose was not reported [31, 33] or treatment duration was unmentioned [31, 39]. These weaknesses in the study designs make them subject to bias and confounding which is also apparent from the RoB assessment. The rarity of the diseases make controlled studies difficult to execute. This would require large multicenter trials and a long follow-up period. The included studies present real-world data and it is perhaps unfair to compare their quality to randomized controlled trials. At the same time the lack of power of these studies to draw clear conclusions on the effectiveness of CA treatment in BASDs illustrates the work to be done. On the one hand, more studies in a controlled setting should be performed. On the other hand, existing data might be better used if data is captured in an independent, multicenter disease registry.

Most studies examined bile acid profiles (urine or serum), liver chemistries and fat-soluble vitamin absorption to determine the effectiveness of CA. However, it is still unclear what the exact correlation is between these outcome measurements and the eventual outcome of the disease. There is still uncertainty on how clinically relevant it is to monitor liver enzymes, while liver enzyme levels may not directly correlate with or predict fibrosis progression. The available publications do not provide an answer on this. Furthermore in the specific case of liver chemistries it is on one hand advised to monitor liver function (AST, ALT, GGT, alkaline phosphatase, bilirubin and INR) to evaluate the effectiveness of CA treatment [21, 22]. However, on the other hand liver function is also monitored to evaluate possible toxicity of CA treatment as CA can also be hepatotoxic [19, 22]. 

As mentioned, several small observational studies have led to authorization of cholic acid as an orphan drug in the EU for 3β-HSD, AKR1D1, CTX, AMACR and CYP7A1 defects, and in the United States for ZSD as well [15, 16, 20]. Despite the heterogeneity with respect to patient population, pathogeneses and clinical and biochemical presentations, the treatment is similar with a recommended dose of 10–15 mg/kg per day [15, 16, 20]. No dose-response studies have been performed to determine this dose. This recommended dose has been based on clinical experience with patient’s response to bile acids [15]. According to the EPAR of Orphacol^®^ and Kolbam^®^ the lowest dose of CA that effectively reduces bile acid metabolites to as close to zero as possible, should be chosen [15, 16]. 

When it comes to the safety and tolerability, CA seems to be tolerated quite well. Most common reported adverse events were diarrhea, elevated bilirubin levels and elevated liver chemistries (including hepatic failure). Most of the reported adverse events were not thought to be serious and were resolved after decreasing CA dose.

Despite treatment, disease progression and hepatic failure were also amongst the more often reported SAEs. These terms are interchangeable for bile acid synthesis effects as hepatic failure is one of the major signs of disease progression. While treatment failed to prevent these manifestations, one may argue that in these cases the disease was too advanced for treatment to be effective. This is also described for some of the deceased patients, mentioning liver failure and end-stage liver disease at the time of diagnosis for these patients. The question however is, whether an earlier start of treatment would have prevented or delayed liver disease or disease progression.

In 2018 the National Institute for health and Care Excellence (NICE) performed a clinical evidence review of CA for treating inborn errors of primary bile acid synthesis [43]. Four studies were included in the NICE review, covering a time period of 1985 to 2017. Three included studies have also been included in this review [14, 37, 39]. The other included studies in this review were either excluded from the NICE review (due to a set inclusion criteria of a minimum number of included people), or weren’t published yet at the time the NICE review was performed. No definite conclusions were drawn in the NICE review on the effectiveness or safety of CA treatment for BASD. The main conclusions were similar even though more (recent) studies have been included in this review; randomized controlled studies and dose-response studies are missing, and methods were not always clearly defined. The studies are subject to bias and confounding due to the lack of controls, open-label nature of the studies, double counting of participants due to overlap in some studies, and missing data and statistical analyses. Moreover, in the NICE review concerns were expressed about the use of the same outcome measures for all the studied BASD deficiencies as they present with different clinical features. Overall there is limited data on starting CA treatment in adults, and there is no experience of CA treatment in the elderly population. No guidelines or policies on managing inborn errors of primary bile acid synthesis due to 3β-HSD, AKR1D1, CTX, AMACR and CYP7A1 deficiencies with cholic acid treatment were drafted based on NICE’s review. This review has a broader scope as no exclusion was set on the number of people included in the studies.

## Authors’ conclusion

The available data to date is insufficient to draw definite conclusions on the effectiveness of CA treatment in patients with bile acid synthesis defects. This is worrying because CA is an authorized treatment for these diseases under exceptional circumstances, with annual re-assessment of safety and efficacy data. However, this data does not seem to have been published openly. More studies in controlled setting (with inclusion and exclusion criteria, pre-defined outcome measurements and definitions) are required in order to evaluate the effectiveness of CA as a treatment for these diseases. At the same time, existing real-world data might be put to better use if captured in an independent disease registry.

## Electronic supplementary material

Below is the link to the electronic supplementary material.

Supplementary Material 1

## Data Availability

All data collected and analysed during this study are included in this article and its supplementary information file.

## Abbreviations

BASDs: Bile Acid Synthesis Defects
CA: Cholic Acid
3β-HSD: 3β-Hydroxy-Δ^5^-C_27_-Steroid oxidoreductase
5β-reductase: Δ^4^-3-oxosteroid-5β-reductase
AE: Adverse Event
AF: Alkaline phosphatase
ALT: Alanine Transaminase
AMACR: α-methylacyl-CoA Racemase
aPTT: Activated Partial Thromboplastin Clotting Time
AST: Aspartate aminotransferase
CDCA: Chenodeoxycholic Acid
CRD: Centre for Reviews and Dissemination
CTX: Cerebrotendinous xanthomatosis, sterol 27-hydroxylase deficiency
CYP7A1: Cholesterol 7α-hydroxylase
DHCA: Dihydroxycholestanoic Acid
eGFR: Estimated Glomerular Filtration Rate
EU: European Union
GCT: Giant Cell Transformation
GGT: Gamma-Glutamyl Transferase
HCC: Hepatocellular Carcinoma
LLN: Lower Level of Normal
METAVIR: Meta-Analysis of histological data in Viral hepatitis
NA: Not Applicable
NICE: National Institute for health and Care Excellence
PICOS: Population, Intervention, Comparison, Outcomes and Study design
PT: Prothrombin Time
RCTs: Randomized Controlled Trials
RoB: Risk of Bias
ROBINS-I: Risk Of Bias IN Non-randomised Studies of Interventions - assessment tool
SAE: Serious Adverse Events
SD (score): Standard Deviation (score)
SEDs: Single Enzyme Deficiencies in bile acid synthesis
TCA: Taurocholic Acid
THCA: Trihydroxycholestanoic Acid
UDCA: Ursodeoxycholic acid
ULN: Upper Level of Normal
ZSD: Zellweger Spectrum Disorders
