# Supplementary material for: The clinical and biochemical effectiveness and safety of cholic acid treatment for bile acid synthesis defects: a systematic review
Source: Orphanet J Rare Dis. 2024 Dec 19;19:466. doi: 10.1186/s13023-024-03449-7 (PMC11657003; doi:10.1186/s13023-024-03449-7)
Supplement: Supplementary file 1 — Supplementary Material 1 [file 13023_2024_3449_MOESM1_ESM.docx]

## Pubmed

Database(s): Ovid MEDLINE(R) and Epub Ahead of Print, In-Process & Other Non-Indexed Citations. Search restricted to studies with human subjects. Language restricted to English and German. No date restriction.

Search Strategy: October 21 2020

| **Search** | **Query 21-10-2020** | **Results** |
| --- | --- | --- |
| #3 | Search: **#1 AND #2** Sort by: **Most Recent** | [450](https://pubmed.ncbi.nlm.nih.gov/?term=%231+AND+%232&sort=date) |
| #2 | Search: **Bile acid synthesis defect, congenital, 1 [Supplementary Concept] OR 3-beta-hydroxy-delta-5-c27-steroid-oxidoreductase-deficiency OR Bile acid synthesis defect, congenital, 2 [Supplementary Concept] OR "delta 4-3-oxosteroid 5-beta-reductase deficiency" OR Xanthomatosis, cerebrotendinous [MeSH] OR "CTX" OR "Cerebrotendinous Xanthomatos*" OR ("Cerebral" AND "Cholesterinos*" )OR "Bogaert Scherer Epstein" OR "sterol 27-hydroxylase deficiency" OR Alpha methylacyl coa racemase deficiency [supplementary concept] OR (("Alpha-methylacyl-CoA racemase" OR "2-methylacyl-CoA racemase" OR "AMACR" OR "Cholesterol 7-alpha-hydroxylase" OR "CYP7A1") AND ("deficien*" OR "defect")) OR "Cholesterol 7-alpha-Hydroxylase/deficiency"[Mesh] OR Zellweger syndrome [MeSH] OR ("Zellweger*" AND "syndrome") OR ("Zellweger*" AND "spectrum") OR "ZSD" OR "ZS " OR "ZSS" OR "cerebrohepatorenal syndrome" OR "cerebro-hepato-renal syndrome" OR "peroxisomal biogenesis disorder*" OR "PBD" OR "PBDs" OR ("congenital" AND "bile acid" AND "synthesis" AND "defect*") OR ("progressive" AND "familial" AND "intrahepatic" AND "cholestasis") OR "PFIC" OR "BASD*"** Sort by: **Most Recent** | [22,789](https://pubmed.ncbi.nlm.nih.gov/?term=Bile+acid+synthesis+defect%2C+congenital%2C+1+%5BSupplementary+Concept%5D+OR%0D%0A3-beta-hydroxy-delta-5-c27-steroid-oxidoreductase-deficiency++OR%0D%0A%0D%0ABile+acid+synthesis+defect%2C+congenital%2C+2+%5BSupplementary+Concept%5D+OR%0D%0A%E2%80%9Cdelta+4-3-oxosteroid+5-beta-reductase+deficiency%E2%80%9D+OR%0D%0A%0D%0AXanthomatosis%2C+cerebrotendinous+%5BMeSH%5D+OR+%0D%0A%E2%80%9CCTX%E2%80%9D++OR%0D%0A%E2%80%9CCerebrotendinous+Xanthomatos%2A%E2%80%9D++OR%0D%0A%28%E2%80%9CCerebral%E2%80%9D+AND+%E2%80%9CCholesterinos%2A%E2%80%9D++%29OR%0D%0A%E2%80%9CBogaert+Scherer+Epstein%E2%80%9D++OR%0D%0A%E2%80%9Csterol+27-hydroxylase+deficiency%E2%80%9D+OR%0D%0A%0D%0AAlpha+methylacyl+coa+racemase+deficiency+%5Bsupplementary+concept%5D+OR%0D%0A%28%28%E2%80%9CAlpha-methylacyl-CoA+racemase%E2%80%9D++OR+%E2%80%9C2-methylacyl-CoA+racemase%E2%80%9D+OR+%E2%80%9CAMACR%E2%80%9D+OR+%E2%80%9CCholesterol+7-alpha-hydroxylase%E2%80%9D+OR+%E2%80%9CCYP7A1%E2%80%9D%29+AND+%28%E2%80%9Cdeficien%2A%E2%80%9D+OR+%E2%80%9Cdefect%E2%80%9D%29%29+OR%0D%0A%22Cholesterol+7-alpha-Hydroxylase%2Fdeficiency%22%5BMesh%5D++OR%0D%0A%0D%0AZellweger+syndrome++%5BMeSH%5D+OR%0D%0A%28%E2%80%9CZellweger%2A%E2%80%9D+AND+%E2%80%9Csyndrome%E2%80%9D%29+OR%0D%0A%28%E2%80%9CZellweger%2A%E2%80%9D+AND+%E2%80%9Cspectrum%E2%80%9D%29+OR%0D%0A%E2%80%9CZSD%E2%80%9D+OR%0D%0A%E2%80%9CZS+%E2%80%9D+OR%0D%0A%E2%80%9CZSS%E2%80%9D+OR+%0D%0A%E2%80%9Ccerebrohepatorenal+syndrome%E2%80%9D+OR%0D%0A%E2%80%9Ccerebro-hepato-renal+syndrome%E2%80%9D+OR%0D%0A%E2%80%9Cperoxisomal+biogenesis+disorder%2A%E2%80%9D+OR%0D%0A%E2%80%9CPBD%E2%80%9D+OR%0D%0A%E2%80%9CPBDs%E2%80%9D+OR%0D%0A%0D%0A%28%E2%80%9Ccongenital%E2%80%9D+AND+%E2%80%9Cbile+acid%E2%80%9D+AND+%E2%80%9Csynthesis%E2%80%9D+AND+%E2%80%9Cdefect%2A%E2%80%9D%29++OR%0D%0A%28%E2%80%9Cprogressive%E2%80%9D+AND+%E2%80%9Cfamilial%E2%80%9D+AND+%E2%80%9Cintrahepatic%E2%80%9D+AND+%E2%80%9Ccholestasis%E2%80%9D%29++OR%0D%0A%E2%80%9CPFIC%E2%80%9D+OR%0D%0A%E2%80%9CBASD%2A%E2%80%9D+%0D%0A&sort=date) |
| #1 | Search: **Cholic acid [MeSH] OR "cholic acid*" OR "cholate*" OR "sodium cholate*" OR "cholalic acid" OR "cholsaure " OR "cholbam" OR "orphacol"** Sort by: **Most Recent** | [19,172](https://pubmed.ncbi.nlm.nih.gov/?term=Cholic+acid+%5BMeSH%5D+OR+%0D%0Achenodeoxycholic+acid++%5BMeSH%5D+OR%0D%0A%E2%80%9Ccholic+acid%2A%E2%80%9D++OR%0D%0A%E2%80%9Ccholate%2A%E2%80%9D+OR%0D%0A%E2%80%9Csodium+cholate%2A%E2%80%9D+OR+%0D%0A%E2%80%9Ccholalic+acid%E2%80%9D+OR%0D%0A%E2%80%9Ccholsaure+%E2%80%9D+OR+%0D%0A%E2%80%9Ccholbam%E2%80%9D+OR+%0D%0A%E2%80%9Corphacol%E2%80%9D+OR%0D%0A%E2%80%9CCDCA%E2%80%9D+OR%0D%0A%28%28%E2%80%9Cchenodeoxycholic%E2%80%9D+OR+%E2%80%9Cursodeoxycholic%E2%80%9D+OR+%E2%80%9Ctaurodeoxycholic%E2%80%9D+OR+%E2%80%9Ctauroursodeoxycholic%E2%80%9D+OR+%E2%80%9Cglycodeoxycholic%E2%80%9D+OR+%E2%80%9Cchenic%E2%80%9D+OR+%E2%80%9Cchenique%E2%80%9D%29+AND+%E2%80%9Cacid%E2%80%9D%29+OR%0D%0A%E2%80%9Chenohol%E2%80%9D+OR%0D%0A%E2%80%9Cchenodiol%E2%80%9D+OR%0D%0A%E2%80%9Cchenodeoxycholate%E2%80%9D+OR%0D%0A%E2%80%9Cchenofalk%E2%80%9D+OR%0D%0A%E2%80%9Cchenophalk%E2%80%9D+OR%0D%0A%E2%80%9Cchenix%E2%80%9D+OR%0D%0A%E2%80%9Cdeoxycholylglycine%E2%80%9D+OR%0D%0A%28%E2%80%9Cglycine%E2%80%9D+AND+%E2%80%9Ddeoxycholate%E2%80%9D%29+OR%0D%0A%E2%80%9Cglycodeoxycholate%E2%80%9D+OR%0D%0A%E2%80%9Cdeoxycholyltaurine%E2%80%9D+OR%0D%0A%28%E2%80%9Cdeoxycholate%E2%80%9D+AND+%E2%80%9Ctaurine%E2%80%9D%29+OR%0D%0A%E2%80%9Ctaurodeoxycholate%E2%80%9D%0D%0A&sort=date) |

The following synonyms do not return any hits:

- CBAS1
- BASD1
- BASD2
- Bile acid synthesis defect, congenital, 4 [Supplementary Concept]
- Liver disease-retinitis pigmentosa-polyneuropathy-epilepsy syndrome
- Colalin
- Cholalin
- kolbam
- Gallodesoxycholic Acid
- Acid, Gallodesoxycholic
- Quenocol
- Quenobilan

MeSH

**Cholic Acids**

[Cholic Acid](https://www.ncbi.nlm.nih.gov/mesh/68019826)

[Cholates](https://www.ncbi.nlm.nih.gov/mesh/68020355) +

[Dehydrocholic Acid](https://www.ncbi.nlm.nih.gov/mesh/68003685)

[Deoxycholic Acid](https://www.ncbi.nlm.nih.gov/mesh/68003840)

[Chenodeoxycholic Acid](https://www.ncbi.nlm.nih.gov/mesh/68002635) +

[Glycodeoxycholic Acid](https://www.ncbi.nlm.nih.gov/mesh/68006002) +

[Taurodeoxycholic Acid](https://www.ncbi.nlm.nih.gov/mesh/68013657) +

[Ursodeoxycholic Acid](https://www.ncbi.nlm.nih.gov/mesh/68014580)

[Glycocholic Acid](https://www.ncbi.nlm.nih.gov/mesh/68006000)

[Glycodeoxycholic Acid](https://www.ncbi.nlm.nih.gov/mesh/68006002) +

[Lithocholic Acid](https://www.ncbi.nlm.nih.gov/mesh/68008095)

[Taurolithocholic Acid](https://www.ncbi.nlm.nih.gov/mesh/68013658)

[Taurocholic Acid](https://www.ncbi.nlm.nih.gov/mesh/68013656)

[Taurodeoxycholic Acid](https://www.ncbi.nlm.nih.gov/mesh/68013657) +

[Taurolithocholic Acid](https://www.ncbi.nlm.nih.gov/mesh/68013658)
